# Aging With HIV in Nigeria: A Narrative Review of Multimorbidity and Healthcare Access Challenges

**DOI:** 10.1002/hsr2.71184

**Published:** 2025-08-19

**Authors:** Sunkanmi Folorunsho, Beulah Suleman

**Affiliations:** ^1^ Department of Sociology University of Nebraska‐Lincoln Lincoln Nebraska USA

**Keywords:** chronic disease, healthcare access, HIV, multimorbidity, Nigeria, older adults

## Abstract

**Background and Aims:**

The population of older adults living with HIV (OALHIV) in Nigeria is growing, bringing forth a dual challenge of chronic comorbidities and barriers to adequate healthcare. This narrative review aims to synthesize empirical evidence on multimorbidity, treatment access, and systemic healthcare gaps affecting OALHIV in Nigeria.

**Methods:**

A structured narrative review was conducted using peer‐reviewed articles and institutional reports published between 2005 and 2024. Searches were performed across PubMed, Google Scholar, African Journals Online (AJOL), ScienceDirect, and WHO and UNAIDS databases. Eligible studies focused on individuals aged 50 years and older living with HIV in Nigeria and addressed at least one of the following themes: chronic disease burden, healthcare access, mental health, or socioeconomic vulnerabilities.

**Results:**

Findings show that multimorbidity is common among OALHIV; yet, HIV programs rarely integrate screening for non‐communicable diseases. Gender disparities further shape access: older women often face economic hardship, while older men are more likely to delay care‐seeking. Depression and social isolation are frequently reported but rarely addressed within existing care frameworks. Structural barriers such as poverty and limited social protection hinder consistent ART adherence and engagement in care.

**Conclusion:**

OALHIV in Nigeria face intersecting clinical, economic, and psychosocial burdens that are under‐recognized in the current HIV response. There is an urgent need for integrated service delivery models, mental health integration, and sustainable financing strategies. Future research should prioritize longitudinal and qualitative studies on aging with HIV, especially concerning multimorbidity, stigma, and structural inequality.

## 1 Introduction

Over the past two decades, a growing body of literature has examined the intersection of HIV and aging in sub‐Saharan Africa [1, 2]. In Nigeria, this demographic shift is increasingly evident, with people aged 50 and older representing approximately 13% of all people living with HIV (PLWH) as of 2022 [3]. Although this proportion is lower than in countries such as South Africa or the United States, it still reflects a growing subpopulation of older adults living with HIV (OALHIV), largely due to the success of antiretroviral therapy (ART) in extending life expectancy [2]. As individuals live longer with HIV, they increasingly face age‐related health challenges, including the rising burden of multimorbidity. In this context, multimorbidity refers to the presence of two or more chronic health conditions that co‐occur with HIV [4]. These conditions often include hypertension, type 2 diabetes, cardiovascular disease, chronic obstructive pulmonary disease (COPD), and arthritis [5, 6].

Despite progress in treatment coverage, the healthcare system in Nigeria remains predominantly oriented toward younger PLWH, often overlooking the distinct medical, psychosocial, and structural needs of older adults. Research indicates that OALHIV in Nigeria experience disproportionately high rates of multimorbidity. For instance, one study in Oyo State found that 78.4% of older PLWH had multiple chronic conditions, compared with 52.1% of HIV‐negative peers [5]. Additional studies have identified common clusters of metabolic conditions such as obesity, diabetes, and cardiovascular disease [1]. These comorbidities not only reflect the physiological effects of aging but are also shaped by long‐term ART exposure and broader social determinants of health.

HIV‐related care outcomes among older adults differ substantially from those of younger cohorts. Older adults are more likely to receive a late diagnosis, experience poorer immune reconstitution despite ART, and face higher mortality rates [3]. A cohort study from Jos reported that although 74.8% of older adults achieved viral suppression, their CD4 count recovery was significantly lower than that of younger individuals [3]. These clinical outcomes are compounded by gaps in the routine screening of non‐communicable diseases (NCDs), which remain insufficiently integrated into HIV care programs. A typical HIV care visit in Nigeria, particularly in public health facilities, focuses on ART dispensation, basic symptom assessment, and periodic viral load monitoring. However, these visits often exclude more comprehensive assessments related to aging and multimorbidity.

Gender disparities also contribute significantly to healthcare access challenges among OALHIV. In general, women in Nigeria are more likely to seek healthcare services but encounter structural barriers such as financial dependence, out‐of‐pocket medication costs, and caregiving responsibilities [7]. Equally, men are less likely to seek early care and often present with advanced disease and lower CD4 counts [5]. These patterns reflect broader gender norms in the country, where men's health‐seeking behavior is constrained by sociocultural perceptions of masculinity, while women's access to healthcare is limited by economic and logistical factors [6].

Beyond clinical concerns, the psychosocial burden of aging with HIV is considerable. Depression, anxiety, and social isolation are common among OALHIV, yet mental health services remain underdeveloped and poorly integrated into HIV care systems [8, 9]. A recent study indicated that over 30% of OALHIV in Nigeria report depressive symptoms, though few have access to appropriate psychological support [9]. HIV‐related stigma worsens these issues, as many OALHIV experience social rejection, diminished roles within families, and a loss of financial independence [6]. Economic insecurity is a persistent theme, with many OALHIV lacking formal pensions, struggling with out‐of‐pocket health expenses, and facing neglect rooted in stigma and discrimination [8].

While the broader landscape of aging with HIV encompasses a wide array of medical and social dimensions, this review is focused more narrowly. Specifically, this article synthesizes existing empirical research from Nigeria to explore three interconnected themes: the prevalence and clinical impact of multimorbidity among OALHIV; healthcare access challenges, including deficiencies in screening and continuity of care; and the structural and social determinants, such as stigma, gender inequality, and economic hardship, that shape treatment outcomes and well‐being. By centering the review on these core areas, this article aims to inform policy, identify service delivery gaps, and guide future research in geriatric HIV care within the Nigerian context.

## 2 Methodology

### 2.1 Review Design and Rationale

We employed a structured narrative review to synthesize empirical evidence on the double burden of chronic disease and healthcare access challenges among OALHIV in Nigeria. A narrative review was selected due to its capacity to integrate diverse research designs and provide contextual insight into health and social issues. Additionally, to enhance methodological rigor, we followed the best‐practice framework outlined by Siddaway, Wood, and Hedges, which recommends transparency in literature identification, study selection, and thematic synthesis for narrative reviews [10]. This approach was particularly appropriate for a field characterized by fragmented evidence and complex social determinants.

### 2.2 Review Objectives

Our narrative review was guided by four key objectives. First, it aimed to synthesize empirical findings on the prevalence and burden of multimorbidity among OALHIV in Nigeria, with attention to patterns of coexisting chronic conditions. Second, it sought to evaluate the available evidence on healthcare access, treatment outcomes, and the structural or programmatic gaps within HIV service delivery systems for this aging population. Third, the review examined the role of social determinants, including gender inequality, HIV‐related stigma, and economic insecurity, in shaping health outcomes and healthcare utilization among older adults with HIV. Finally, it aimed to identify existing knowledge gaps and propose evidence‐informed policy directions to improve care delivery and long‐term outcomes for aging individuals affected by HIV in Nigeria.

### 2.3 Eligibility Criteria

We included studies that met several criteria to ensure contextual applicability. First, regarding the population of interest, studies were eligible if they focused on individuals aged 50 years and older living with HIV. This age threshold aligns with UNAIDS and global HIV‐aging research guidelines and reflects a pragmatic definition of “older” in HIV research, acknowledging the accelerated aging often observed among PLWH.

The review prioritized studies conducted in Nigeria. However, studies from other sub‐Saharan African countries were also considered if they provided meaningful comparative insights applicable to the Nigerian context, particularly regarding health systems, care infrastructure, or social determinants of health.

Eligible study designs included empirical studies employing quantitative, qualitative, or mixed‐methods approaches. Policy reports with clearly described methodologies were also included, as well as gray literature from authoritative health organizations such as UNAIDS, the World Health Organization (WHO), and the Nigerian Federal Ministry of Health. In contrast, opinion pieces, editorials lacking empirical data, and clinical or biomedical studies that did not address aging‐specific or social dimensions were excluded to maintain thematic coherence.

Studies had to address at least one of the following core themes to be included: the burden of chronic disease or multimorbidity among older adults with HIV; access to HIV care and treatment outcomes; mental health, stigma, and social vulnerability; or structural and policy‐related barriers to care. Additionally, to ensure both historical depth and contemporary relevance, only studies published between 2005 and 2024 were included. This period captures key developments in ART rollout and healthcare policy evolution in Nigeria. Additionally, all studies had to be published in English to ensure accessibility and interpretive clarity.

### 2.4 Search Strategy

We conducted a comprehensive literature search across multiple academic and institutional databases to identify relevant studies. The databases searched included PubMed, Google Scholar, African Journals Online (AJOL), ScienceDirect, Web of Science, and official repositories from institutions such as UNAIDS and the WHO. Search terms were developed using Boolean logic to ensure both breadth and specificity. Combinations of keywords used in the search included “HIV AND aging AND Nigeria,” “Older adults AND HIV AND chronic disease,” “Multimorbidity AND HIV AND sub‐Saharan Africa,” “HIV care access AND older people,” “HIV‐related stigma AND elderly,” and “Geriatric care AND ART adherence AND Nigeria.” These terms were selected to capture literature that intersects aging, HIV, multimorbidity, healthcare access, and social determinants of health in the Nigerian or comparable regional contexts.

To supplement the database searches, a snowballing strategy was applied. This involved reviewing the reference lists of studies identified through the initial search to locate additional sources that may not have appeared in the primary database results. This dual approach ensured a more exhaustive retrieval of relevant literature.

### 2.5 Selection Process

**Figure 1:**
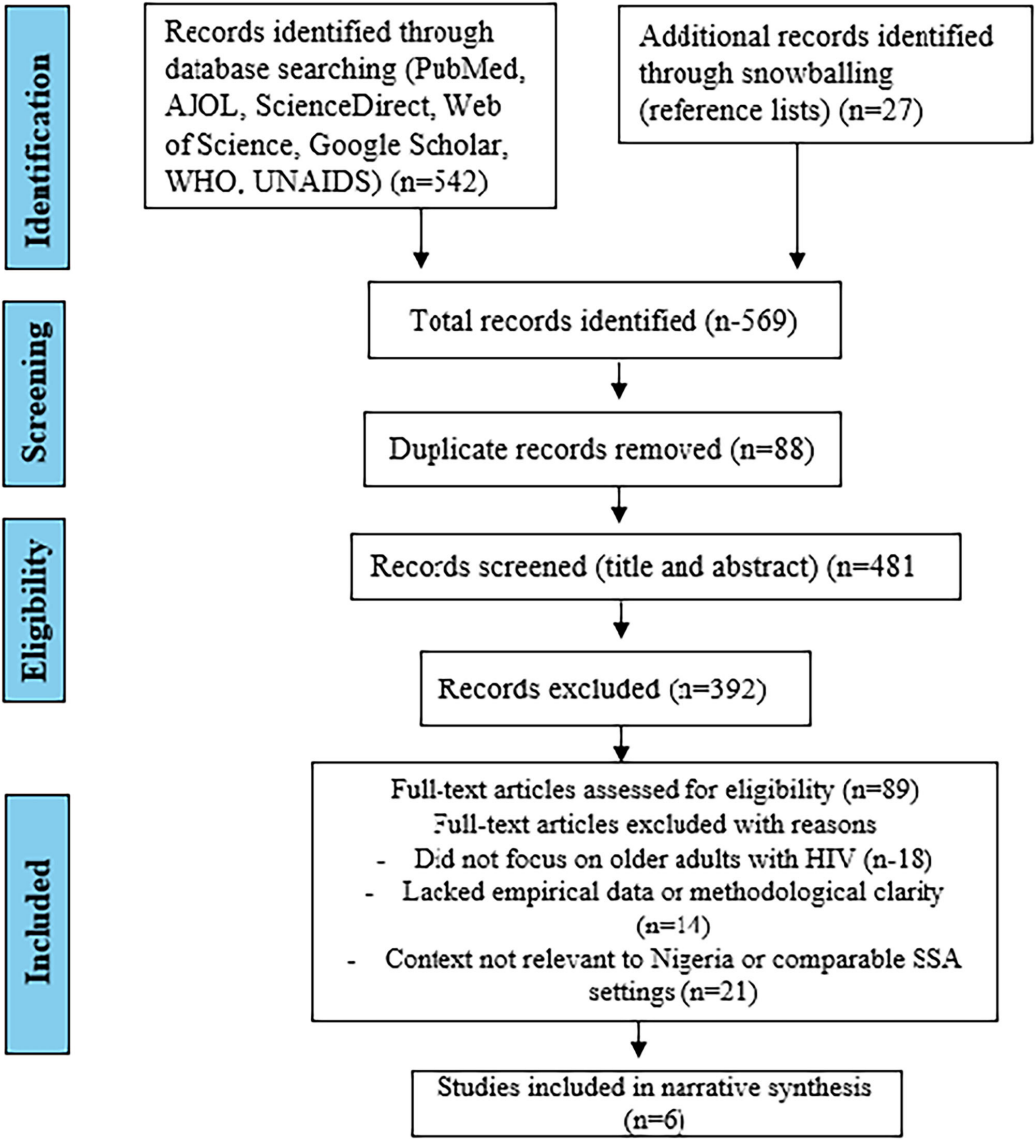
PRISMA flow diagram of study selection process.

Our study selection followed the Preferred Reporting Items for Systematic Reviews and Meta‐Analyses (PRISMA) guidelines [11]. Duplicates were removed, and the remaining records were screened based on titles and abstracts. Full‐text reviews were conducted for studies that appeared to meet the inclusion criteria. Discrepancies in inclusion were resolved through team discussion. A PRISMA flow diagram illustrating this process is provided in Section 3 (Figure 1).

### 2.6 Data Extraction and Synthesis

Key variables extracted from each study included the author names, year of publication, and geographic location of the research. Additional information captured included the study design and sample characteristics such as participant age, gender distribution, and regional representation.

Health outcomes were recorded with particular attention to the presence of comorbid conditions and mental health concerns among OALHIV. Metrics related to HIV care and treatment such as ART adherence, retention in care, and viral suppression were also documented. Reported barriers to healthcare access, including stigma, economic insecurity, and limitations within the health system, were analyzed. Where applicable, policy recommendations or implications for healthcare practice were noted as part of the synthesis. Finally, all extracted data were narratively synthesized and grouped thematically in alignment with the review's objectives. Descriptive statistics including sample sizes and prevalence estimates were retained when available to enhance interpretive clarity and contextual understanding.

Table 1 included the operationalization of key terms for easy understanding.

**Table 1:** Operationalization of key terms.

Key term	Operational definition
Multimorbidity	Presence of two or more chronic conditions in an individual, measured through self‐reports, medical records, or clinical diagnoses.
Chronic disease burden	Impact of chronic illnesses on health, functionality, and quality of life, quantified by the number, type, and severity of conditions.
Older adults living with HIV (OALHIV)	Individuals aged 50 years or older diagnosed with HIV and receiving ART in Nigeria.
Opportunistic infections (OIs)	HIV‐associated infections occurring due to weakened immunity, such as oral candidiasis, chronic diarrhea, tuberculosis, and pneumonia.
Antiretroviral therapy (ART) adherence	Extent to which individuals follow prescribed ART regimens, assessed through self‐reports, pharmacy refill data, or viral suppression rates.
Viral suppression	Reduction of HIV viral load to an undetectable level (< 200 copies/mL) as a result of consistent ART use.
Healthcare access	Ability of OALHIV to receive medical care, including screenings for chronic diseases, ART management, and mental health support.
Gender disparities in healthcare access	Differences in healthcare‐seeking behaviors, ART initiation, and treatment adherence between older men and women living with HIV.
Mental Health (depression and anxiety in OALHIV)	Presence of psychological distress, particularly depression and anxiety, in older adults with HIV.
Stigma and social isolation	Negative social attitudes and discrimination faced by OALHIV, leading to exclusion from family and community support systems.
Economic hardship	Financial constraints limiting access to healthcare, medications, or stable livelihood.
Quality of life (QoL) in OALHIV	Multidimensional measure of well‐being, considering physical health, mental health, financial stability, and social relationships.

## 3 Results

### 3.1 Study Selection and PRISMA Flow Summary

A total of 569 records were identified through database searching (*n* = 542) and snowballing from reference lists (*n* = 27). After removing 88 duplicates, 481 unique records were screened by title and abstract. Of these, 392 were excluded due to irrelevance or duplication. The remaining 89 full‐text articles were assessed for eligibility. After full‐text review, 83 studies were excluded for the following reasons: the study did not focus on older adults with HIV (*n* = 18); lacked empirical data or methodological clarity (*n* = 14); or were not relevant to the Nigerian context or comparable sub‐Saharan African settings (*n* = 21). In total, six studies met the inclusion criteria and were included in the final narrative synthesis (Figure 1).

### 3.2 Contextual Interpretation of Aging, HIV, and Health System Challenges in Nigeria

Table 2 highlights the demographic and health system context within which older adults with HIV in Nigeria navigate care. With over 9% of PLWH aged 50 and above, and multimorbidity affecting more than a third of older adults, the intersection of aging and HIV demands integrated service delivery. Yet, gaps in health insurance coverage, specialist geriatric services, and chronic disease screening illustrate persistent systemic barriers. These structural constraints directly inform the healthcare access and treatment challenges described in the reviewed studies.

**Table 2:** Contextual overview of aging and HIV in Nigeria.

Indicator	Description	Data source/Statistic
Population aging	Estimated proportion of adults aged ≥ 50 years in Nigeria	~10.2% [12]
HIV prevalence (general population)	Overall adult HIV prevalence in Nigeria	1.4% [13]
HIV prevalence among adults aged ≥ 50	HIV prevalence in older adults	Estimated 5.3% (NACA, 2022); up to 8.2% in regional cohorts
Common chronic conditions	Most prevalent comorbidities among older PLWH	Hypertension, Type 2 diabetes, osteoarthritis, COPD [5, 14]
Multimorbidity rate	Proportion of older PLWH with ≥ 2 chronic conditions	42%–67% in selected studies [5, 6]
Routine screening coverage	Proportion of older PLWH receiving NCD screening (e.g., BP, glucose)	< 40% receive consistent screening [4]
ART coverage	ART access among older PLWH	> 90% nationally enrolled, but care quality varies by region [4]
Healthcare access barriers	Key barriers to care for older adults with HIV	Long wait times, poor transportation, cost of medications, lack of geriatric‐specialized care [15]
Mental health integration	Availability of mental health support in ART clinics	Limited to major urban centers; < 15% of facilities provide integrated care [15]

### 3.3 Study Characteristics and Main Findings

The six included studies were conducted in diverse Nigerian regions, including Ibadan, Jos, Nnewi, Akwa Ibom & Cross River, and one national‐level survey (Table 3). All studies focused on individuals aged 50 years and older, consistent with global standards for defining aging in the context of HIV. Sample sizes ranged widely, from 186 participants in a comparative clinical study to over 16,000 in a multi‐state retrospective analysis. Four studies utilized retrospective cohort or epidemiological designs; one used a prospective cohort, and another employed a cross‐sectional approach. Recruitment settings were predominantly HIV clinics or ART centers, with one study leveraging national survey data. Across the studies, common chronic conditions included hypertension, diabetes, opportunistic infections, and reduced immune recovery. Despite variations in setting and design, all studies pointed to the intersection of HIV, aging, and multimorbidity as a significant public health concern in Nigeria.

**Table 3:** Characteristics of selected studies and main findings.

Author (year)	Location	Study design	Population and setting	Sample size	Age (mean, SD) or range	HIV & associated chronic conditions	Main findings	Implications
Obimakinde et al. [5]	Ibadan, Nigeria	Comparative cross‐sectional	Older PLWH (≥ 60 years) attending Infectious disease and geriatric clinics	186 (62 HIV + , 124 HIV−)	Mean: 63.9 (HIV + ), 68.1 (HIV‐)	Higher multimorbidity, lower CD4 count, increased non‐HIV conditions (hypertension, diabetes)	PLWH had more chronic MM (2.0 vs. 1.3, *p* = 0.004), earlier onset (4.7 vs. 9.6 years, *p* = 0.003), and lower quality of life (82.7 vs. 86.2, *p* = 0.002).	HIV treatment programs must integrate multimorbidity care for aging PLWH in Nigeria.
Akinyemi et al. [6]	Ibadan, Nigeria	Retrospective epidemiological	Older adults (≥ 50 years) newly enrolled for HIV care at ART clinic	2075 older adults	Mean: 50+ (not specified)	46.6% had opportunistic infections (oral candidiasis, chronic diarrhea, peripheral neuropathy); lower CD4 linked to higher OI risk	Opportunistic infections prevalent in 46.6% of older adults; CD4 < 350 associated with 3.12x higher risk (CI: 2.29‐4.25).	Older adults require early detection and better management of opportunistic infections.
Abimiku et al. [8]	National (Nigeria)	Population‐based survey	Adults (15‐64 years) on ART from national HIV/AIDS survey	1322 PLWH	Median: 39.31 years (IQR: 31.47–47.63)	80.9% had viral suppression, older age was linked to better VLS	Older age (45–54) linked to higher odds of viral suppression (aOR = 2.81, 95% CI: 1.14–6.90).	Targeted interventions needed for young PLWH to improve medication adherence and viral suppression.
Agaba et al. [3]	Jos, Nigeria	Retrospective cohort	Older adults (≥ 50 years) initiating ART at Jos University Teaching Hospital	8352 (643 aged ≥ 50)	≥ 50 years (age range not specified)	Older adults had better viral suppression (74.8% vs. 69.6% younger adults) but lower immune recovery	Older adults achieved better viral suppression but had lower immune reconstitution compared to younger patients.	Older adults may require enhanced immune recovery support alongside ART regimens.
Akpan et al. [14]	Akwa Ibom & Cross River, Nigeria	Retrospective analysis	Older PLWH ( ≥ 50 years) from 154 health facilities and community sites	16,420 older adults	≥ 50 years	Higher hypertension prevalence (9.6%), with suboptimal screening impacting long‐term care	Retention rate at 96.4%, viral suppression 99.0%; however, 9.6% had hypertension, males had lower rates (8% vs. 11.1%).	Need for integrated hypertension screening and management in HIV care for older adults.
Stephen et al. [7]	Nnewi, Nigeria	Prospective cohort	Older adults (≥ 50 years) attending HIV Counseling and Testing (HCT)	4384 older adults	≥ 50 years (age range not specified)	HIV prevalence: 8.2%; more common in females; higher in 50–59 age group than 60+	HIV prevalence: 8.2% among ≥ 50, with highest rate among 50‐59 age group; more prevalent in females.	Routine HIV testing for older adults should be expanded for early detection and treatment linkage.

### 3.4 Double Burden of Chronic Disease in Older Adults With HIV

Across the reviewed studies, chronic disease burden emerged as a prominent concern among OALHIV. Obimakinde et al. found that HIV‐positive older adults had significantly higher multimorbidity (2.0 vs. 1.3 conditions; *p* = 0.004) compared with HIV‐negative counterparts, as well as earlier onset of chronic diseases such as hypertension and diabetes [5]. Similarly, Akpan et al. reported that 9.6% of OALHIV across 154 sites presented with hypertension, with a gender differential in prevalence (11.1% in women vs. 8.0% in men) [14]. Akinyemi et al. documented that 46.6% of newly enrolled older ART patients experienced opportunistic infections, with a threefold increase in risk among those with CD4 counts below 350 cells/mm³ [6]. These findings underscore the complex interaction between HIV and age‐related comorbidities in Nigeria.

### 3.5 Healthcare Access and Treatment Challenges

Access to effective HIV and chronic disease care remains inconsistent for older adults in Nigeria. Although ART programs have improved viral suppression rates, other health indicators reveal persistent gaps. Agaba et al. found that older ART recipients had better viral suppression (74.8%) than younger adults (69.6%), but lower immune reconstitution, suggesting that viral suppression alone may not translate into improved functional health in older age [3]. Abimiku et al. similarly showed that older adults (aged 45–54) had higher odds of viral suppression (adjusted odds ratio = 2.81; 95% CI: 1.14–6.90) compared with younger age groups [8].

However, most studies did not explore specific structural barriers to care, such as healthcare staffing, long travel times, or service delivery design. Only Akpan et al. provided indirect insight by noting limited screening for hypertension and inadequate integration of NCD care into existing HIV services [14]. Delayed diagnosis also emerged as a concern. Stephen et al. reported an HIV prevalence of 8.2% among adults aged 50 and older, highest among those aged 50–59, which may suggest ongoing gaps in early detection and targeted testing services for older adults [7].

### 3.6 Social and Economic Barriers to HIV Care

Although socioeconomic factors were not the primary focus of most included studies, several findings indirectly highlighted their relevance. Akpan et al. found gender‐based differences in hypertension prevalence, suggesting that care‐seeking behavior and health service utilization may differ by sex [14]. While direct measurements of economic hardship, stigma, or mental health were not common in the reviewed studies, prior literature indicates that older Nigerian adults (particularly women) may experience financial barriers, out‐of‐pocket medication costs, and reduced access to transportation and caregiving support.

## 4 Discussion

OALHIV in Nigeria face a dual burden of managing HIV alongside age‐associated chronic conditions, a reality more accurately described as multimorbidity, which refers to the coexistence of two or more chronic diseases. While ART has significantly improved survival rates, the Nigerian healthcare system remains inadequately equipped to manage the broader spectrum of aging‐related health challenges in this population. Our review of six empirical studies reveals a growing prevalence of conditions such as hypertension, diabetes, and opportunistic infections among aging PLWH. These health burdens are compounded by the limited integration of geriatric care into existing HIV programs, insufficient routine screening practices, and widespread socioeconomic vulnerability, all of which compromise long‐term outcomes.

Findings from Obimakinde et al. and Akpan et al. emphasize that multimorbidity among OALHIV not only occurs frequently but also manifests earlier than in their HIV‐negative peers, with hypertension and diabetes particularly prominent [5]. Likewise, Akinyemi et al. identified a persistent burden of opportunistic infections in older PLWH, often linked to late HIV diagnosis and poor CD4 recovery [6]. Although studies by Agaba et al. and Abimiku et al. report encouraging viral suppression rates, they also reveal that immune reconstitution remains suboptimal, suggesting that ART alone is insufficient to meet the comprehensive health needs of aging PLWH in Nigeria [3, 8]. Notably, only two studies examined barriers to accessing care for multimorbidity, and none addressed the roles of stigma or mental health comorbidities, which are critical gaps that limit the applicability and depth of current evidence. Furthermore, the lack of longitudinal or qualitative studies constrains our understanding of how health systems respond over time to the evolving needs of this demographic.

In contrast, other African contexts such as South Africa and Malawi offer more advanced approaches to managing multimorbidity in aging HIV‐positive populations. South Africa's Integrated Chronic Disease Management (ICDM) model has demonstrated success in co‐delivering HIV, diabetes, and hypertension care at the primary healthcare level, leading to improved ART retention and blood pressure control. Zimbabwe's Friendship Bench initiative, which incorporates mental health support into HIV services, has shown reductions in depressive symptoms among PLWH [16]. These models provide valuable and contextually relevant insights that Nigeria can draw upon, particularly by adopting strategies like task‐shifting, community‐based follow‐up, and the integration of psychosocial support within routine HIV care.

### 4.1 Policy and System‐Level Recommendations

To address the needs of PLWMM (People Living with Multimorbidity) among OALHIV, healthcare systems must prioritize the integration of HIV services with chronic disease management. Evidence from Akpan et al. points to significant gaps in hypertension screening—despite a 9.6% prevalence among study participants [14]. This calls for policy reform within Nigeria's National HIV/AIDS Strategic Framework to embed routine screening and management of NCDs (e.g., hypertension, diabetes) within ART programs. As ART clinics already serve as a routine point of contact, they offer a strategic entry point for expanded geriatric services.

Sustainable financing is also essential. As donor support continues to decline, Nigeria must increase its domestic health budget and expand the coverage of the National Health Insurance Authority (NHIA) to include care for multiple chronic conditions among older PLWH. Recent reductions in global HIV funding programs, such as the United States President's Emergency Plan for AIDS Relief (PEPFAR) and USAID, have underscored the urgency of developing locally sustained financing mechanisms [13]. Nigeria can draw lessons from Ghana's National Health Insurance Scheme (NHIS), which offers older adults access to both HIV and chronic disease services [17]. Adopting a similar integrated financing model could alleviate financial hardship and improve treatment adherence among older populations.

### 4.2 Strengthening Community‐Based and Mental Health Services

Our review revealed limited attention to socioeconomic barriers, yet multiple studies implied that clinic‐based care may be inadequate, particularly in rural regions [7]. Expanding home‐based services using Community Health Extension Workers (CHEWs) can address mobility constraints and reduce loss to follow‐up. Ethiopia's experience with CHEWs in TB and HIV treatment follow‐up offers a practical precedent, showing reduced default rates and improved ART adherence [18].

Mental health remains a critical but neglected dimension of multimorbidity. While none of the reviewed studies directly examined depression or stigma, prior evidence suggests that aging PLWH in Sub‐Saharan Africa face higher rates of psychological distress due to ageism and HIV‐related stigma [19]. Nigeria could replicate Zimbabwe's *Friendship Bench* or Kenya's *SHARE* intervention, which integrate low‐cost mental health counseling into ART programs [20].

### 4.3 Clarifying Solutions Versus Research Needs

Clarifying the distinction between immediate solutions and future research needs is vital for guiding both policy implementation and academic inquiry. In terms of service integration, a key priority is incorporating routine screening for NCDs such as hypertension and diabetes within existing ART clinics. This is a feasible step that requires minimal infrastructural overhaul. However, it should be complemented by longitudinal research to track the progression and outcomes of multimorbidity among OALHIV in Nigeria. In community‐based care, expanding the role of CHEWs and home‐based ART support could bridge access gaps, particularly in underserved regions. There is also a pressing need for qualitative studies that explore the lived experiences of aging PLWH navigating fragmented care systems. On the financial front, expanding the NHIA to include chronic illness coverage would ease the economic burden on older adults. Research into the cost‐effectiveness of integrated HIV‐NCD services could inform scalable models. Mental health services demand immediate attention. Implementing task‐shifted counseling programs, such as the Friendship Bench model, could address the growing psychological needs of OALHIV [21]. Further research is needed on stigma‐reduction strategies tailored to this population. Lastly, public education campaigns involving community and religious leaders can reduce both HIV‐related stigma and ageism. These efforts would benefit from community‐based participatory research that investigates how stigma uniquely impacts older adults with HIV across diverse Nigerian contexts.

### 4.4 Youth‐to‐Older Age Continuum

Planning sustainable HIV care systems requires a forward‐looking, lifespan approach that recognizes the trajectory from youth to older adulthood. Investing in the health and well‐being of younger adults living with HIV is not only a matter of immediate public health concern but also a strategic foundation for managing future aging cohorts. As highlighted by Abimiku et al. [18], younger PLWH in Nigeria exhibit significantly lower rates of viral suppression compared with their older counterparts. This disparity reflects systemic gaps in early HIV diagnosis, consistent ART adherence, and access to psychosocial support services for younger populations. Strengthening interventions at this earlier stage, such as expanding youth‐friendly testing centers, introducing peer‐based adherence programs, and integrating mental health services, can mitigate long‐term health complications and prevent the early onset of comorbid conditions. Moreover, fostering health literacy and resilience among younger PLWH through targeted education campaigns can promote long‐term engagement with care systems. Supporting younger cohorts today will help Nigeria proactively shape a healthier and more stable population of OALHIV in the future. This approach can reduce the burden of multimorbidity and lower long‐term healthcare costs. A lifespan perspective is essential for building a comprehensive HIV response that anticipates demographic shifts and prioritizes continuity of care.

### 4.5 Limitations and Implications

As a narrative review, our study is inherently limited by the scope, quality, and heterogeneity of the included literature. Most of the studies were cross‐sectional in design, which restricts the ability to infer causality or track changes over time. There was a notable absence of longitudinal studies that could offer deeper insights into the progression of multimorbidity or the long‐term effectiveness of integrated care models. Additionally, key domains such as mental health, HIV‐related stigma, and the lived experiences of older adults were underrepresented in the existing evidence base. Despite these limitations, the review synthesizes available data to identify consistent themes and critical policy gaps. The recommendations outlined are grounded in repeated patterns across studies and further informed by best practices and comparative insights from other sub‐Saharan African countries. By mapping the limitations of the current research landscape, this review highlights areas where targeted investment and focused inquiry could substantially enhance the quality of care for older OALHIV in Nigeria.

## Author Contributions

**Sunkanmi Folorunsho:** conceptualization, methodology, formal Analysis, writing – original draft, visualization. **Beulah Suleman:** supervision, writing – review and editing, validation.

## Conflicts of Interest

The authors declare no conflicts of interest.

## Transparency Statement

The lead author Sunkanmi Folorunsho affirms that this manuscript is an honest, accurate, and transparent account of the study being reported; that no important aspects of the study have been omitted; and that any discrepancies from the study as planned (and, if relevant, registered) have been explained.

## Data Availability

The authors have nothing to report.
